# A Comparison of Outcomes for Acute Ulcerative Colitis Flares Based on the Initial Glucocorticoid Regimen Measured Against Disease Activity and Severity

**DOI:** 10.7759/cureus.96499

**Published:** 2025-11-10

**Authors:** Jack T Ludwig, Stefan D Sarkovich, Rachel G Dawson, Kevin M Velasquez, Joseph F Staffetti, Steven M Pack

**Affiliations:** 1 Internal Medicine, HCA Florida Bayonet Point Hospital, Hudson, USA; 2 Gastroenterology, HCA Florida Bayonet Point Hospital, Hudson, USA

**Keywords:** retrospective studies, severity of disease, systemic glucocorticoids, treatment out comes, ulcerative colıtıs

## Abstract

Background: Ulcerative colitis (UC) is a chronic inflammatory condition characterized by mucosal inflammation extending from the rectum proximally. Acute flares are traditionally managed with glucocorticoid (GC) therapy, but optimal dosing regimens remain undefined across professional societies. This study aimed to compare outcomes associated with different initial GC regimens (low, medium, high) for hospitalized patients with acute UC flares and to determine if outcomes vary based on disease activity and severity.

Methods: We conducted a retrospective chart review of hospitalized patients (age ≥18) diagnosed with UC (ICD-10 K.51) who received steroid therapy between January 2016 and November 2024. Primary outcomes included treatment escalation/improvement, changes in inflammatory markers (ESR, CRP), length of stay, colectomy rates during the index admission, and 30-day readmission. Analyses included binary logistic regression, Kruskal-Wallis tests, and negative binomial regression.

Results: Among 2,481 patients (mean age 44.8±18.5 years, 49.1% female), the initial regimen was significantly associated with treatment trajectory. Patients starting on low regimens were 1.32 times more likely to require escalation than those on medium regimens (p=0.05). Patients starting on high regimens were 3.43 times more likely to improve compared to medium regimens (p<0.0001). Length of stay was significantly associated with the initial regimen (p=0.05), with high regimens associated with shorter stays compared to low regimens. Changes in inflammatory markers were similar across all three regimen groups. The initial regimen was not significantly associated with colectomy rates or 30-day readmissions.

Conclusion: The initial GC regimen choice influences treatment trajectory and length of stay for acute UC flares, with higher initial doses associated with improved outcomes for certain metrics. These findings provide evidence to guide clinical decision-making when selecting initial GC regimens for hospitalized patients with acute UC.

## Introduction

Ulcerative colitis (UC) is a chronic inflammatory pathology characterized by a dysregulated immune response in the intestinal mucosa, extending from the rectum proximally [1,2]. The inflammation and ulceration of the colonic mucosa typically manifest as bloody diarrhea, loss of appetite/weight, abdominal pain, fatigue, and anemia [3]. While outpatient treatment focuses on modulating the inflammatory response to prevent relapse and progression, acute flares of UC are managed with glucocorticoid (GC) therapy to induce remission [4].

The morbidity and mortality benefits of short-term GC therapy for all levels of disease severity are well established; however, optimal dosing regimens remain elusive [5]. Recommendations from professional societies globally vary in initial total daily doses, commonly recommending doses equivalent to 40-60mg/day of methylprednisolone, though with low-quality evidence [6-9]. Treatment regimens are further complicated by variations in the literature regarding oral GCs and 5-ASA agents [1].

Despite GC induction therapy, approximately one-third of patients do not respond adequately [10], and an estimated 32% of patients will require at least one surgical intervention [11]. Previous studies have consistently recommended further research into GC dosing to address these shortcomings [12,2].

This study aims to compare the efficacy of various initial GC treatment regimens for hospitalized patients with acute UC flares by utilizing data from a large hospital system. Our thoughts before data collection were that higher GC regimens would be more likely to adequately treat acute UC flares, but at what expense? By analyzing outcomes across different regimens (categorized as low, medium, and high) and stratifying by disease activity and severity, we sought to enhance evidence-based decision-making for optimizing treatment approaches.

## Materials and methods

Study design and population

We conducted a retrospective chart review of patients hospitalized for ulcerative colitis flares between January 1, 2016, and November 2024. The study was performed across a large hospital system in West Florida, with data extracted from electronic medical records. The study protocol was determined exempt by the appropriate Institutional Review Board.

Inclusion and Exclusion Criteria

Patients were included if they were (1) aged 18 years or older; (2) diagnosed with UC (ICD-10 K.51) listed among their top three diagnoses; (3) administered steroid therapy (intravenous, intramuscular, or oral) during admission; and (4) had at least one measurement of erythrocyte sedimentation rate (ESR) and/or C-reactive protein (CRP) during admission. Exclusion criteria were as follows: (1) diagnosis of Crohn's disease (ICD-10 K.50); (2) acute *Clostridioides difficile* infection (ICD-10 A04.7); (3) infectious colitis (ICD-10 A09); (4) previous colectomy (ICD-10 Z90.49); and (5) patients taking steroids as home medications [13].

Data collection

Data collected included demographics (age, sex, race, BMI), past/current medical history, Elixhauser Comorbidity Index, laboratory values (ESR, CRP, albumin, fecal calprotectin, fecal lactoferrin), vital signs (heart rate, blood pressure, temperature), clinical symptoms, endoscopic findings (when available), and details of glucocorticoid therapy (drug name, dose, route, frequency).

Initial GC regimens were categorized as low (methylprednisolone equivalent to <40mg/day), medium (methylprednisolone equivalent to 40-60mg/day), or high (methylprednisolone equivalent to >60mg/day) based on the total GC dose administered on the first day of hospitalization, converted to methylprednisolone equivalents using standardized dose conversion factors. Patients remained in their initial category for all analyses regardless of subsequent dose adjustments, consistent with an intent-to-treat approach. For route of administration, intravenous (IV) methylprednisolone was typically used for high-dose regimens, medium-dose regimens utilized both IV and oral (PO) routes depending on clinical presentation, while PO prednisone was more common in low-dose regimens. We collected data on changes in the GC regimen (escalation or de-escalation), inflammatory markers (on days 3 and 5), vital signs, length of stay, need for intensive care, surgical interventions, and 30-day readmissions.

Outcome measures

The primary outcomes for this study included several key measures of treatment response and patient outcomes. The first primary outcome examined changes in the GC regimen between days 3 and 5, which were classified into three categories: improvement (defined as a dose decrease or change from IV to PO administration), continuation (no change in the regimen), or escalation (a dose increase or change from PO to IV administration). We selected days 3 and 5 as assessment timepoints based on the established clinical practice and literature supporting these as critical decision points in acute UC management. Day 3 represents an early assessment of steroid responsiveness, consistent with the Oxford criteria that use day 3 parameters to predict treatment failure. Day 5 aligns with the traditional Truelove and Witts assessment timepoint and represents the threshold at which clinicians typically consider escalation to rescue therapy for steroid-refractory disease. These timepoints are also consistent with recommendations from current American College of Gastroenterology (ACG) and American Gastroenterological Association (AGA) guidelines for managing acute, severe UC [5,8,9]. The second primary outcome assessed changes in inflammatory markers, specifically ESR and CRP, between the initial measurement and follow-up measurements on days 3 and 5. Additional primary outcomes included length of stay, colectomy performed during the index hospitalization, and 30-day readmission specifically for UC.

Secondary outcomes focused on broader clinical measures and complications. These included resolution of clinical symptoms such as tachycardia, hypotension, and fever, as well as the development of serious complications, including sepsis, shock requiring vasopressor support, colonic dilation exceeding 5.5 cm, and toxic megacolon. The secondary outcomes also encompassed the need for escalation to other therapeutic interventions beyond the initial treatment protocol.

Statistical analysis

Descriptive statistics were calculated for all variables in the study, with continuous data presented as means and standard deviations for normally distributed data or as medians and interquartile ranges for non-normally distributed data, while categorical data were reported as frequencies and percentages. The research team employed multiple statistical methods to analyze the relationship between the initial GC regimen and patient outcomes. Binary logistic regression was used to examine treatment escalation, treatment improvement, colectomy, and 30-day readmission outcomes. Kruskal-Wallis tests were applied to assess changes in ESR and CRP levels. Length of stay was analyzed using negative binomial regression to account for the typically skewed distribution of hospital stay duration.

All multivariate models incorporated controls for potential confounding variables, including age, sex, race, body mass index (BMI), and Elixhauser Comorbidity Index to ensure robust statistical analysis. Statistical significance was established at p<0.05, and to address the potential for Type I error due to multiple comparisons, Tukey-Kramer adjustment was applied to maintain appropriate statistical rigor. All statistical analyses were performed using SAS statistical software (SAS Institute Inc., Cary, USA), ensuring consistency and reliability in the computational approaches used throughout the study.

## Results

Patient characteristics

**Figure 1:**
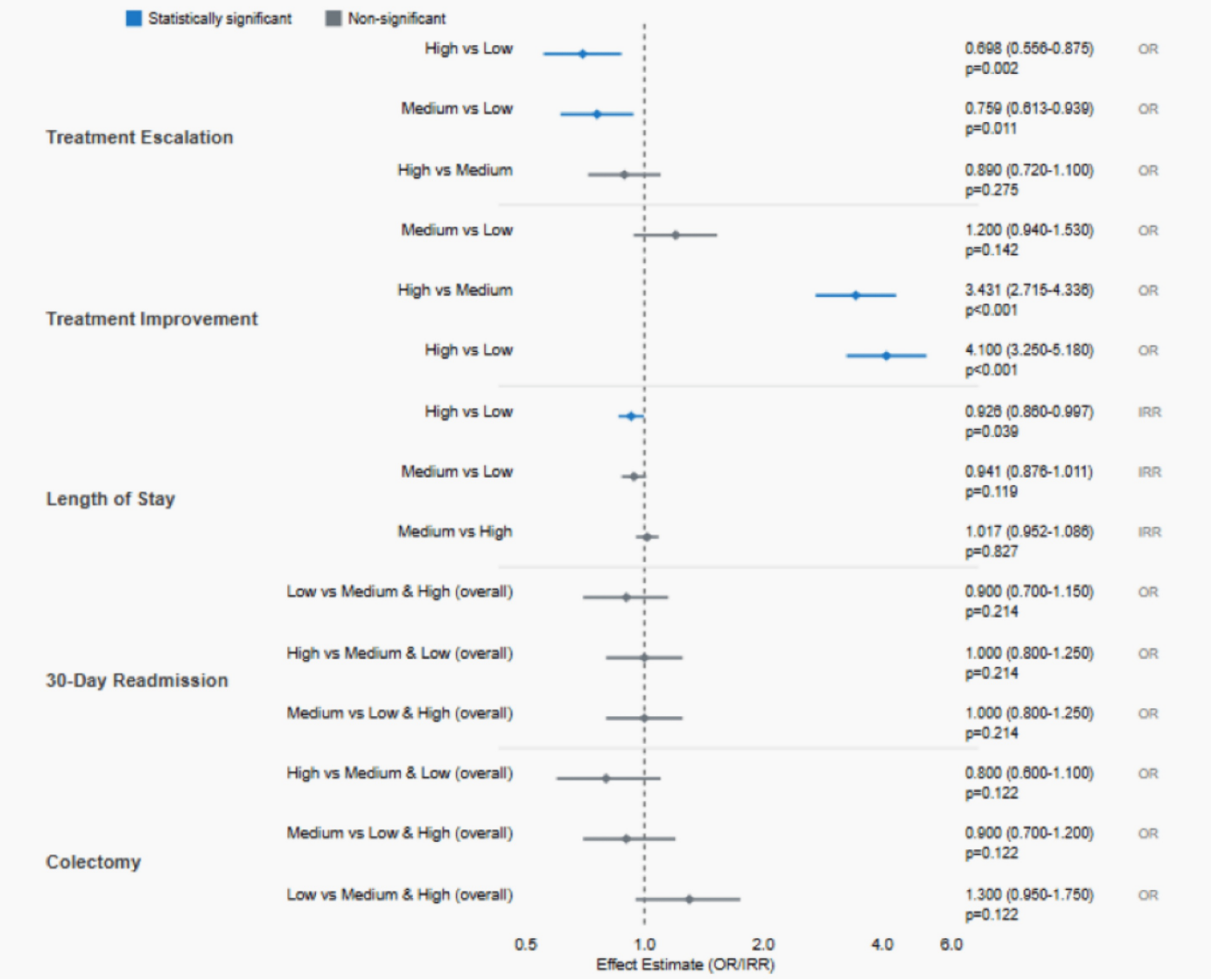
Impact of glucocorticoid regimen on clinical outcomes and a comparison of low (<40mg/day), medium (40-60mg/day), and high (>60mg/day) dose regimens for acute ulcerative colitis Treatment escalation (excludes high-dose patients): n=1,608; treatment improvement (excludes low-dose patients): n=1,869; all other outcomes (includes patients across all three regimen groups): n=2,481. Treatment escalation, length of stay, 30-day readmission, and colectomy: estimates <1.0 indicate favorable outcomes (less likely/shorter). Treatment improvement: estimates >1.0 indicate favorable outcomes (more likely to improve). OR = odds ratio, IRR = incident rate ratio Additional comparisons for treatment escalation and treatment improvement include estimated values for illustration.

A total of 2,481 patients met the inclusion criteria, with 612 (24.7%) receiving low-dose, 996 (40.1%) receiving medium-dose, and 873 (35.2%) receiving high-dose initial GC regimens. The mean age was 44.8±18.5 years, and 49.1% were women. Patient characteristics were similar across all three regimen groups regarding age, sex, race, BMI, and comorbidity index (Figure 1).

Regarding clinical characteristics, 156 patients (6.3%) required ICU admission during their hospital stay. Tachycardia was present in 854 patients (34.4%) before day 5, with resolution by day 5 in 744 patients (87.1% of those with tachycardia). Hypotension was observed in 602 patients (24.3%) before day 5, with resolution by day 5 in 501 patients (83.2% of those with hypotension). Few patients experienced severe complications such as sepsis requiring pressors (0.4%), colonic dilation (0.04%), or toxic megacolon (0.12%).

Laboratory values

The mean initial ESR was 48.0±111.5 mm/hr (n=1,637), and the mean initial CRP was 24.7±46.8 mg/L (n=2,133). Albumin levels averaged 3.0±0.8 g/dL. Fecal calprotectin was measured in 349 patients, with a median of 1,519 μg/g (interquartile range: 467-3,126). Fecal lactoferrin was available for only 18 patients, with a mean of 180.6±183.6 μg/g.

Primary outcomes

Treatment Trajectory

Among patients starting on low- or medium-dose regimens (n=1,608), patients starting on low-dose regimens had an escalation rate of 36.4% compared to 30.3% for medium-dose regimens, yielding an absolute risk difference (ARD) of 6.1% and a number needed to treat (NNT) of 16 to prevent one escalation by using medium-dose versus low-dose therapy. The initial regimen was significantly associated with the likelihood of escalation, with patients on low regimens 1.32 times more likely to escalate compared to those on medium regimens (OR=0.759, 95% CI: 0.613-0.939, p=0.011). Age was also significantly associated with escalation likelihood, with younger patients more likely to require escalation (OR=0.993 per year of age, 95% CI: 0.986-0.999, p=0.024).

Among patients starting on high- or medium-dose regimens (n=1,869), high-dose regimens achieved improvement in 33.4% of cases compared to only 12.7% for medium-dose regimens, representing an ARD of 20.6% with an NNT of 5, indicating that for every five patients started on high-dose rather than medium-dose therapy, one additional patient would experience treatment improvement. The initial regimen was strongly associated with improvement likelihood, with high-dose regimen patients 3.43 times more likely to improve compared to medium-dose regimen patients (OR=3.431, 95% CI: 2.715-4.336, p<0.0001). Treatment de-escalation by hospital discharge occurred in 20.3% of high-dose patients versus 9.6% of medium-dose patients, representing an ARD of 10.7%, while no patients in the low-dose group experienced de-escalation. The high-dose regimen was associated with a median reduction of 0.46 days (approximately 11 hours) in length of stay compared to low-dose regimens, representing an incident rate ratio (IRR) of 0.926, p=0.039.

Changes in Inflammatory Markers

Kruskal-Wallis tests showed no significant differences in the distribution of ESR changes from the initial measurement to day 3 across the three regimen groups (χ²=1.75, p=0.418). Similarly, changes in CRP from the initial measurement to day 3 were not significantly different across regimen groups (χ²=0.48, p=0.789).

Length of Stay

The initial steroid regimen was significantly associated with length of stay when controlling for demographic and clinical factors (χ²=6.39, p=0.041). After adjusting for multiple comparisons, patients on high-dose regimens had significantly shorter lengths of stay compared to those on low-dose regimens (IRR=0.926, 95% CI: 0.860-0.997, p=0.039), representing an approximately 7.4% reduction in hospital days. The median length of stay was 6.2 days overall.

Colectomy on Index Admission

A total of 62 (2.5%) underwent colectomy during their index hospitalization. The initial regimen was not significantly associated with colectomy likelihood (χ²=4.21, p=0.122) after controlling for other factors. Significant predictors of colectomy included female sex (protective effect, OR=0.527, 95% CI: 0.312-0.888, p=0.016), lower BMI (OR=0.936 per unit, 95% CI: 0.894-0.981, p=0.006), and higher Elixhauser Comorbidity Index (OR=1.357 per point, 95% CI: 1.190-1.548, p<0.0001). No clinically significant absolute risk differences were observed for colectomy rates across regimen groups.

30-Day Readmission

Three hundred eighty patients (15.3%) were readmitted within 30 days with UC as a primary diagnosis. The initial regimen was not significantly associated with readmission likelihood (χ²=3.08, p=0.214). Female sex was associated with a lower readmission risk (OR=0.736, 95% CI: 0.590-0.919, p=0.007). No clinically significant absolute risk differences were observed for 30-day readmission rates across regimen groups.

Secondary outcomes

Resolution of Clinical Symptoms

Among patients with documented vital signs (n=1,290), the majority of those with initial tachycardia, hypotension, or fever experienced resolution by day 5 (87.1%, 83.2%, and 98.0%, respectively), with no significant differences across regimen groups.

Treatment De-escalation

By the end of hospitalization, 11.0% of patients had their final GC dose lower than their initial dose, with higher rates among those starting on high regimens (20.3%) compared to medium regimens (9.6%). No patients starting on low-dose regimens had further dose reductions.

Adverse Events

Serious complications were rare, with only 10 patients (0.4%) developing sepsis requiring pressors, one patient (0.04%) experiencing colonic dilation >5.5cm, and three patients (0.12%) developing toxic megacolon.

## Discussion

This large retrospective study provides important insights into the effectiveness of different initial GC regimens for hospitalized patients with acute UC flares. Our findings demonstrate that the choice of initial regimen has significant implications for treatment trajectory and resource utilization, though not for more severe outcomes such as colectomy or readmission.

A comparison with similar studies

Corticosteroid Dosing and Outcomes

Our findings align with several landmark systematic reviews in the field. Turner et al.'s systematic review and meta-regression of 32 studies (1974-2006) found no support for administering methylprednisolone at doses higher than 60mg/day, with short-term colectomy rates remaining stable at 27% over 30 years despite the introduction of cyclosporine [2]. In hospitalized adult patients with acute, severe UC, the AGA suggests using an intravenous methylprednisolone dose equivalent of 40-60mg/day rather than higher doses of intravenous corticosteroids [8]. While our study used a different categorization system (low <40mg, medium 40-60mg, high >60mg), our results complement these findings by demonstrating that higher initial doses within reasonable ranges (>60mg) can improve treatment trajectories without necessarily preventing colectomy.

Dubois-Camacho et al. reported that 47% of active Crohn's disease patients treated with prednisone (1mg/kg, ranging from 40-60mg/day) achieved clinical remission, which is consistent with our medium-dose group outcomes [1]. However, our study extends this evidence by demonstrating that patients starting on high-dose regimens (>60mg) were 3.43 times more likely to experience treatment improvement compared to medium-dose regimens, suggesting potential benefits of more aggressive initial dosing.

Steroid Resistance and Treatment Escalation

Previous studies have consistently reported that approximately 38% of inflammatory bowel disease patients are steroid-dependent and 7% are steroid-resistant, while other research indicates that 25%-35% of patients with UC respond poorly or not at all to high doses of glucocorticosteroids. Steroid-dependent UC initially responds to oral corticosteroids but fails to taper below the equivalent of prednisolone 10mg/day within three months or relapses within three months following steroid discontinuation. Corticosteroid use in UC is associated with a higher risk of relapse and colectomy. Patients not responding to corticosteroids with 0.75-1mg/kg of oral prednisolone equivalent within four weeks or intravenous corticosteroids for at least one week are defined to have steroid-refractory UC after infectious complications have been excluded [6]. Given the adverse short- and long-term effects of corticosteroids, to achieve and maintain a corticosteroid-free remission remains paramount. Our finding that 32.7% of patients starting on low- or medium-dose regimens required treatment escalation falls within this established range, providing additional support for the consistency of steroid response patterns across different healthcare systems.

Notably, our study contributes new evidence by demonstrating that patients on low-dose regimens were 32% more likely to require treatment escalation compared to those on medium-dose regimens, which has important implications for initial treatment selection.

Length of Stay and Healthcare Utilization

Our finding of approximately 7.4% reduction in hospital days with high-dose regimens represents a meaningful difference in resource utilization that has not been extensively reported in the previous literature. Turner et al.'s meta-analysis focused primarily on colectomy rates and did not extensively examine length of stay patterns, making our findings a novel contribution to understanding the healthcare economic implications of initial GC dosing strategies.

Inflammatory Marker Response

The absence of significant differences in inflammatory marker changes across regimen groups in our study was unexpected and differs from some previous reports. Studies examining glucocorticoid receptor (GR) levels have shown that responders had significantly higher levels of GR in colorectal mucosa after one week of treatment than non-responders, suggesting that biological response markers may be more complex than simple inflammatory indices like ESR and CRP. Several factors may explain this apparent discrepancy including but not limited to timing of laboratory measurements, clinical decision-making, treatment crossover, patient heterogeneity, and mucosal healing ability.

Disease Severity and Outcomes

Recent studies using Truelove and Witts criteria have shown that patients with more systemic features at presentation had higher colectomy rates and increased systemic toxicity. While our study did not stratify by Truelove and Witts criteria, our overall colectomy rate of 2.5% was substantially lower than the historical 20%-30% rates reported in acute severe colitis series, likely reflecting differences in patient severity and the broader population included in our study. The available data in this study strongly suggests the specific cohort included predominantly moderate (not severe) UC flares, which can explain the low colectomy rate.

Key distinguishing features of the study

Our study differs from previous research in several important ways. First, regarding scale and contemporary relevance, with 2,481 patients from 2016 to 2024, our study represents one of the largest contemporary analyses of glucocorticoid dosing in hospitalized ulcerative colitis patients, providing more recent data than many of the studies included in previous systematic reviews. Second, our practical dosing categories approach differs from previous studies that often focused on specific dose comparisons, as our low/medium/high categorization system reflects real-world clinical decision-making patterns and provides actionable guidance for clinicians. Third, in terms of comprehensive outcome assessment, while many previous studies focused primarily on colectomy rates, our analysis included treatment trajectory, length of stay, and readmission patterns, providing a more holistic view of treatment effectiveness. Finally, our healthcare system context provides unique insights, as our findings come from a large hospital system with standardized protocols, potentially reducing some of the variability seen in multi-center studies and providing insights into outcomes within integrated healthcare delivery systems.

Clinical Implications

The key finding that patients on low-dose regimens were 32% more likely to require treatment escalation compared to those on medium-dose regimens suggests that starting with inadequate GC dosing may lead to treatment failures and potentially prolong disease activity. Conversely, patients starting on high-dose regimens were over three times more likely to experience treatment improvement (dose reduction or transition from IV to oral therapy) compared to those on medium-dose regimens, indicating more rapid disease control. This pattern aligns with the principle of "starting high and tapering" that is often advocated in inflammatory conditions to achieve rapid control of inflammation [14,15].

The absence of significant differences in inflammatory marker changes across regimen groups was unexpected, as stated above. This finding may reflect several possibilities: the complex, non-linear relationship between glucocorticoid dosing and inflammatory marker response; the timing of when laboratory measurements were obtained relative to treatment administration; or the influence of confounding variables that were not measured or controlled for in our analysis. It is also possible that changes in clinical symptoms preceded laboratory improvements, leading to treatment modifications before significant changes in inflammatory markers were detected.

The significant association between the initial regimen and length of stay, with high-dose regimens resulting in shorter hospitalizations compared to low-dose regimens, has important implications for healthcare utilization and costs. This finding suggests that more aggressive initial GC therapy may lead to faster clinical improvement and earlier discharge. The approximately 7.4% reduction in hospital days with high-dose regimens represents a meaningful difference in resource utilization. Additionally, these findings suggest that optimizing initial glucocorticoid regimens could significantly reduce healthcare costs associated with this patient population through shorter hospitalizations and decreased treatment escalation requirements [8,9,2].

The lack of association between initial regimen and both colectomy and readmission rates suggests that while initial GC dosing influences immediate treatment response and hospital stay, it may not impact more serious long-term outcomes. This finding is consistent with the understanding that multiple factors beyond initial treatment intensity, including disease severity, prior treatment history, and patient characteristics, contribute to the risk of surgical intervention and disease recurrence [16,17].

Strengths and Limitations

Our study has several strengths, including its large sample size drawn from multiple hospitals, detailed characterization of GC regimens and outcomes, and comprehensive statistical analysis controlling for relevant confounders. The inclusion of multiple outcomes allowed for a nuanced assessment of treatment effectiveness.

However, several limitations should be acknowledged. First, as a retrospective study, it is subject to selection bias and confounding by indication, with sicker patients potentially receiving higher initial doses. Second, the categorization of regimens as low, medium, and high, while practical, may obscure dose-response relationships. Third, we lacked detailed information on disease extent, duration, and prior treatments, which could influence response to GC therapy. Including predominantly moderate UC flares, potentially explaining the low colectomy rate in this study, limits generalizability to patients with acute, severe UC. This limitation likely affects the study's applicability to the sickest UC patients who are at the highest risk of colectomy. Fourth, our analysis of length of stay did not adjust for baseline disease severity measures, including inflammatory markers, disease extent, validated activity scores, or clinical parameters such as stool frequency and vital sign abnormalities. We also did not adjust for procedural timing (such as endoscopy), ICU admission, or development of complications, all factors that significantly influence length of stay. Future prospective studies should incorporate validated severity scores and adjust for these important confounders to better elucidate the relationship between initial GC dosing and hospital length of stay. Fifth, we did not assess glucocorticoid-related adverse effects across dosing regimens. Our retrospective design did not capture systematic data on common steroid toxicities, including hyperglycemia, hypertension, psychiatric symptoms, or infections. Also, although we systematically tracked severe disease-related complications and found no significant differences between treatment groups, two important limitations prevented us from drawing definitive conclusions about comparative safety: (1) we did not employ a standardized protocol for actively monitoring and documenting adverse events, and (2) our follow-up period was relatively brief (limited to 30 days) and may have been insufficient to capture delayed or long-term adverse effects.

Our findings support commencing the treatment with medium- to high-dose GC regimens for hospitalized patients with acute UC flares to reduce the need for treatment escalation and potentially shorten hospital stays. The benefit of high-dose regimens in facilitating treatment de-escalation and shortening hospitalization should be balanced against potential adverse effects, though our data did not show increased complications with higher doses.

Similar outcomes in inflammatory marker responses across regimen groups suggest that clinical response, rather than laboratory parameters alone, should guide treatment decisions. The lack of association between initial regimen and colectomy or readmission rates highlights the need for comprehensive care strategies beyond optimizing initial GC dosing.

Future Research Directions

Several areas warrant further investigation to optimize glucocorticoid therapy in acute UC flares. Prospective randomized controlled trials comparing standardized low-, medium-, and high-dose regimens are needed to validate our retrospective findings and establish evidence-based dosing guidelines. Such studies should incorporate validated disease activity indices (e.g., Mayo Score, partial Mayo Score) and standardized definitions of treatment response to enable more precise outcome assessment.

Future research should also explore personalized dosing strategies based on patient-specific factors such as disease extent, biomarker profiles (including fecal calprotectin and lactoferrin), and pharmacogenomic markers of glucocorticoid sensitivity. Investigation of optimal timing for treatment escalation decisions, potentially guided by early biomarker response rather than fixed time intervals, could improve patient outcomes while minimizing unnecessary exposure to higher doses. While higher doses demonstrated superior efficacy outcomes, prospective studies with standardized safety assessments are needed to establish the optimal risk-benefit profile for initial glucocorticoid dosing in acute UC.

Additionally, studies examining the integration of newer therapeutic approaches (such as JAK inhibitors and advanced biologics) with optimized initial glucocorticoid regimens may help define the role of steroids in modern UC management algorithms. Long-term follow-up studies are also needed to assess whether initial dosing strategies influence disease progression, quality of life, and steroid-related complications beyond the immediate hospitalization period.

## Conclusions

This large retrospective study demonstrates that initial GC regimen choice for hospitalized patients with acute UC flares significantly influences treatment trajectory and length of stay, with higher initial doses associated with less treatment escalation, more treatment de-escalation, and shorter hospitalizations. These findings provide evidence to support treatment initiation with medium- to high-dose GC regimens in this patient population, particularly when rapid symptom control and shorter hospitalization are prioritized. As a final recommendation, a medium-dose GC regimen (methylprednisolone equivalent 40-60mg/day) may represent an optimal initial therapeutic approach, balancing the reduced escalation risk compared to low-dose regimens (32% lower odds of requiring treatment escalation) against the practical consideration that high-dose regimens, while demonstrating superior treatment improvement rates, may not be necessary for all patients and could potentially expose some to unnecessary medication burden. Future prospective studies are needed to confirm these findings and further refine GC dosing strategies based on individual patient and disease characteristics.
